# Community-acquired pneumonia work-up in areas where coccidioidomycosis is endemic: Undertested, underdiagnosed, and untreated

**DOI:** 10.1017/ash.2025.314

**Published:** 2025-09-24

**Authors:** Kent Carpenter, Pooja Rangan, Sumit Agarwal, Justin Hayes, Neil Ampel, Jonas Marschall

**Affiliations:** 1 University of Arizona College of Medicine Phoenix; 2 University of Arizona College of Medicine; 3 University of Arizona; 4 University of Arizona College of Medicine – Phoenix

## Abstract

**Background:** The dimorphic fungus Coccidioides is endemic in the Southwestern USA and most commonly causes respiratory infection (“Valley Fever”). While the true community prevalence of this respiratory infection is unknown, experts estimate that 30% of community-acquired pneumonia (CAP) cases in Southern Arizona are due to Coccidioides. We were interested in determining how often patients admitted for CAP are tested for coccidioidomycosis. **Methods:** We identified patients who were admitted to Banner University Medical Center – Phoenix with community-acquired pneumonia from 1/1/2019-6/30/2024 by the ICD-10 code J18.9. Among this patient population, we determined the percentage tested for coccidioidomycosis (via serological test) and the percentage that tested positive. Regarding management, we elicited whether an infectious diseases consultation occurred during the hospitalization and if treatment included the antifungal fluconazole versus ceftriaxone and Azithromycin. **Results:** We identified 9,677 patients admitted with an ICD-10 code J18.9 between 1/1/2019 and 06/30/2024. The mean age (SD) was 60.3 (17.2) years and 56.3% were males. 3,536 (36.5%) patients were tested for coccidioidomycosis, and 389/3,536 (11%) had a positive serology. 14.2% of CAP patients were seen by an ID specialist. Among those with coccidioidomycosis, 56.3% (n=219) were seen by an ID specialist. Only a small fraction (n=974, 10.1%) of all CAP patients received fluconazole. Among the 389 with Valley Fever, 52.2% received fluconazole, while almost 70% were given ceftriaxone and/or azithromycin at any point during the admission. Transfer to the ICU, length of stay and hospital mortality were not significantly different in those with detected coccidioidomycosis versus others. **Conclusions:** In this large observational study in an area endemic for coccidioidomycosis, only 36.5% of those admitted for community-acquired pneumonia were tested for coccidioidomycosis 11% of those who got tested were found to have Valley Fever. Positing a similar coccidioidomycosis prevalence in the remaining 63.5% of CAP patients who were not tested for it, one could extrapolate a total of 676 missed cases based on 11% positive serology rate. To determine the true prevalence of coccidioidomycosis in our region, broader testing should be implemented. Our data also indicate that antifungals are rarely offered for coccidioidal CAP, while unnecessary use of antibacterials for this endemic mycosis is a target for antimicrobial stewardship.

